# A stepwise approach in endovascular treatment for stumpless anterior tibial artery occlusion

**DOI:** 10.1016/j.jvscit.2026.102469

**Published:** 2026-08-24

**Authors:** Tetsuya Nomura, Jun Munakata, Hiroshi Kubota, Naotoshi Wada, Natsuya Keira, Tetsuya Tatsumi

**Affiliations:** Department of Cardiovascular Medicine, Kyoto Chubu Medical Center, Nantan City, Kyoto, Japan

**Keywords:** Endovascular treatment, Critical limb-threatening ischemia, Intravascular ultrasound, Anterior tibial artery occlusion, Retrograde approach

## Abstract

Endovascular treatment of infrapopliteal lesions is challenging. An antegrade approach to the origin of the anterior tibial artery (ATA), which branches sharply from the relatively large popliteal artery with a preserved lumen, can be more challenging than initially expected. Moreover, in cases with ambiguous entry points of the occluded ATA, establishing retrograde access is promising for successful intervention. We present a case of chronic limb-threatening ischemia with an unidentified entry point for ATA occlusion in which stepwise modification of the endovascular treatment strategy was a critical determinant of successful revascularization.

Advancements in techniques and devices for endovascular treatment (EVT) of lower-extremity arterial disease have led to higher revascularization success rates. However, there are several pitfalls in the specific situations of EVT for infrapopliteal lesions.^1^ The anterior tibial artery (ATA) sometimes branches from the popliteal artery (POPA) with marked angulation. Owing to these anatomical features, antegrade vessel preparation can be considerably more challenging than initially anticipated. Moreover, in the cases of stumpless ostial ATA occlusion, establishing a retrograde access is promising for the successful intervention. Informed consent was obtained from the patient for the publication of this case report and the accompanying images.

## Case report

**Fig 1:**
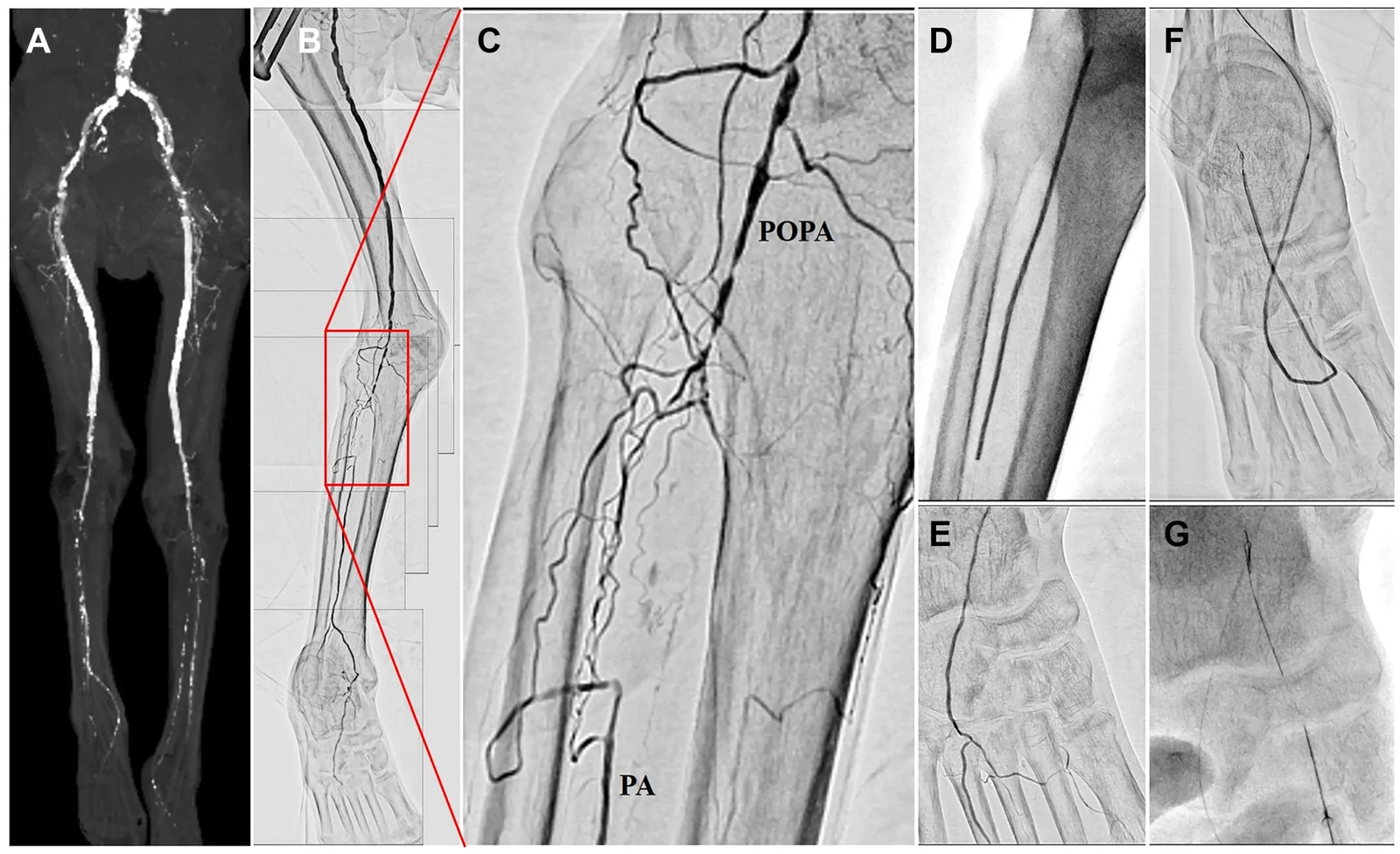
**A,** Computed tomography angiography depicting occlusions of the right anterior tibial artery (ATA) from its ostium (*arrow*) and the tibioperoneal trunk (TPT) with the type IIIA variant anatomy. **B** and **C,** Initial angiography showing diffuse stenoses of the popliteal artery (*POPA*) and occlusions of both the ATA and TPT. **D,** Balloon angioplasty of the occluded TPT. **E,** Angiography of the pedal arch. **F,** Pedal arch angioplasty. **G,** Puncture of the dorsal artery after pedal arch angioplasty. *PA*, Peroneal artery.

A 78-year-old man was admitted to our hospital for the treatment of chronic limb-threatening ischemia in his right lower extremity (Rutherford 5). The wound, ischemia, and foot infection classifications were wound-1, ischemia-3, and foot infection-0, respectively, with a clinical stage 3 and moderate risk of amputation. The ankle-brachial pressure index was not measurable on the right side. Computed tomography (CT) angiography revealed occlusion of the right ATA from the ostium (*arrow*) and tibioperoneal trunk (TPT) with a type IIIA variant anatomy (Fig 1, *A*). We classified it as stage III based on the Global Limb Anatomic Staging System, with femoropopliteal grade 4 and infrapopliteal grade 4 on the right side.^2^ We implemented EVT of the right lower extremity for the purpose of limb salvage.

The patient was administered 100 mg aspirin and 75 mg clopidogrel before catheterization. An ipsilateral common femoral artery access system was established using a 4.5F Parent Plus guide sheath (MEDIKIT). Immediately after guide sheath insertion, an intraarterial bolus of 5000 units of unfractionated heparin was administered. The activated clotting time was controlled to ≥250 seconds during the procedure. Initial angiography indicated diffuse stenosis of the POPA and occlusion of both the ATA and TPT (Fig 1, *B* and *C*). We planned to treat both occlusions considering the distribution of the ulceration on the heel and gangrenes in the second, third, and fifth toes. We could not identify the entry point of the occluded ATA even with intravascular ultrasound (IVUS) guidance. After successfully passing a 0.014-inch Gladius guidewire (ASAHI INTECC) to the patent peroneal artery, the occluded TPT was inflated with a SABER balloon catheter (Cordis; Fig. 1, *D*), leading to guidewire advancement to the lateral plantar artery due to the type IIIA variant anatomy. We advanced a Cruise guidewire (ASAHI INTECC) into the dorsal artery via the pedal arch (Fig 1, *E*). Subsequently, pedal arch angioplasty was performed using a Coyote balloon catheter (Boston Scientific; Fig 1, *F*), which enabled puncture of the dorsal artery to establish a retrograde approach system (Fig 1, *G*).

**Fig 2:**
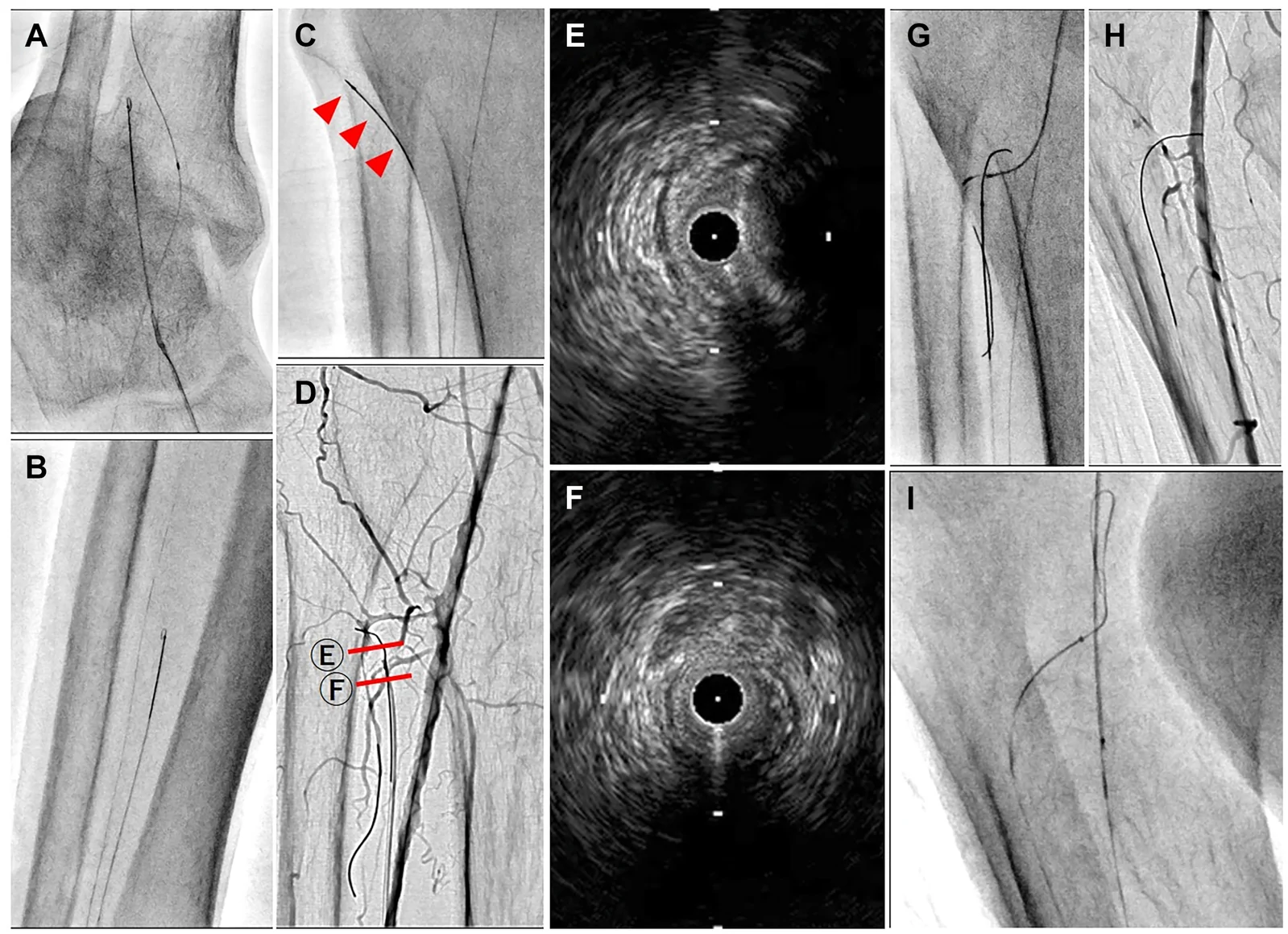
**A,** Microcatheter insertion via the dorsal artery. **B,** Advancement of a small-loop guidewire toward the proximal anterior tibial artery (ATA). **C,** Marked deviation of the retrograde guidewire from the popliteal artery (POPA; *arrowheads*). **D,** Intravascular ultrasound (IVUS) marking at the deviated point from the intraplaque to the subintimal space. **E,** The cross-sectional IVUS image at the points E and F in Fig 2, *D* demonstrating the IVUS transducer in the subintimal space **(E)** and intraplaque area **(F)**, respectively. **G,** Rerouting the retrograde guidewire at the deviated point toward the POPA. **H,** The retrograde guidewire reaching near the POPA. **I,** Successful crossing of the retrograde guidewire.

An Ichibanyari PAD microcatheter (Kaneka Medix) was inserted via the dorsal artery (Fig 2, *A*), and Gladius MG guidewire (ASAHI INTECC) was proximally advanced with a small-loop guidewire technique (Fig 2, *B*). However, the guidewire deviated markedly from the POPA as it gradually approached the proximal side (Fig 2, *C*, *arrowheads*). To identify the point of guidewire deviation from the intraplaque to the subintimal space, we changed the microcatheter to a 3F Parent Plus guide sheath (MEDIKIT) and checked the cross-sectional image using an AnteOwl IVUS (Terumo; Fig 2, *D*). The cross-sectional IVUS image at point E in Fig 2, *D* demonstrates that the IVUS transducer was located in the subintimal space (Fig 2, *E*), in contrast to the intraplaque area at point F in Fig 2, *D* and *F*). We then rerouted a Halberd guidewire (ASAHI INTECC) at the guidewire-deviated point confirmed with IVUS toward the POPA, relying on the tactile feeling of the guidewire (Fig 2, *G* and *H*), and finally succeeded in retrograde guidewire crossing (Fig 2, *I*).

**Fig 3:**
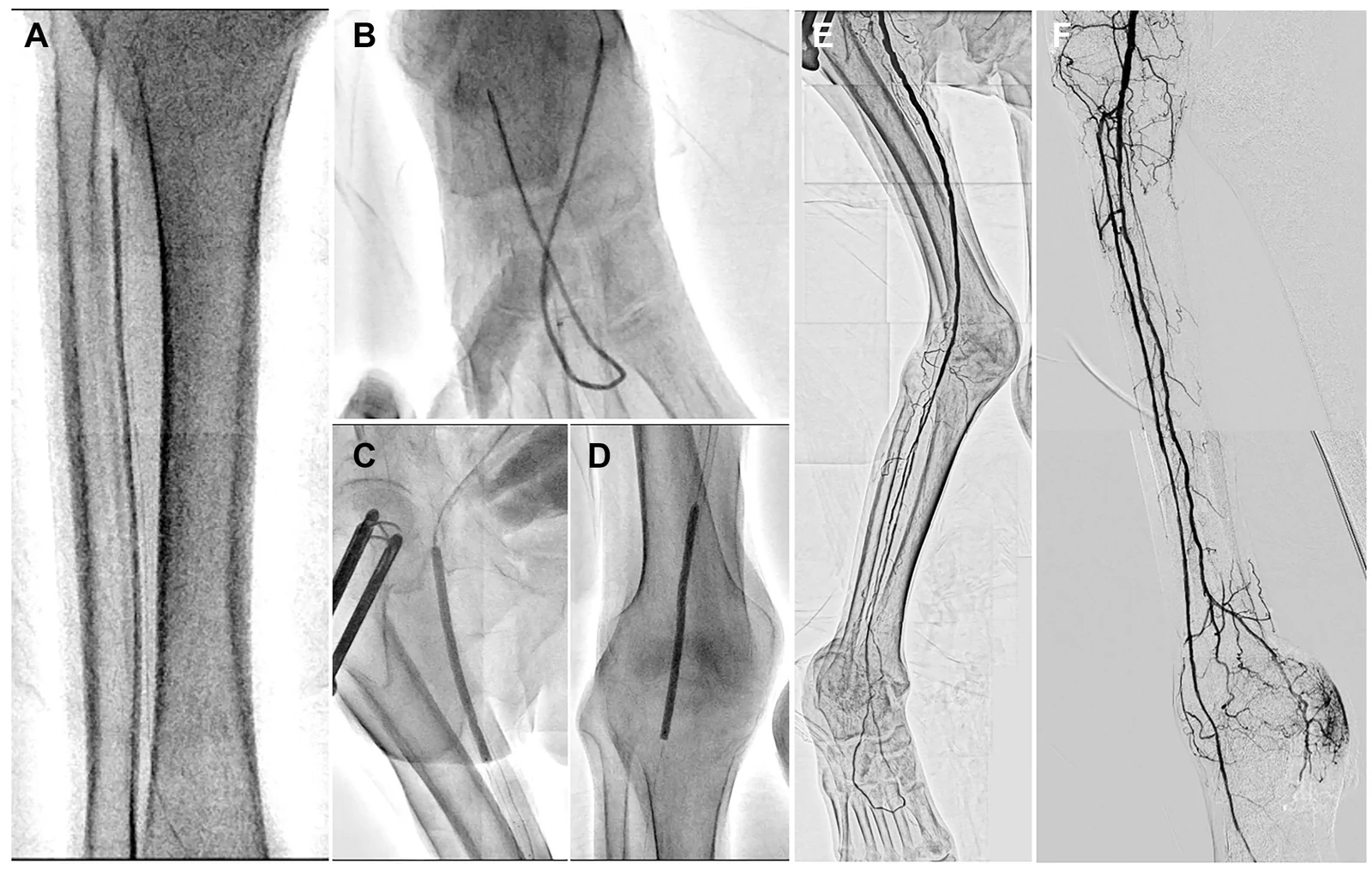
**A,** Balloon angioplasty of the anterior tibial artery (ATA). **B,** Retrograde guide sheath removal with balloon compression from the inside of the pedal arch. Dilation of two drug-coated balloon catheters at the proximal superficial femoral artery (SFA) **(C)** and popliteal artery (POPA) **(D)**, respectively. **E** and **F,** Final angiography depicting good vascular patency and favorable blood flow in the right lower extremity.

The retrograde guidewire was externalized via the antegrade Parent Plus guide sheath, and a CROSSLEAD Tracker guidewire (ASAHI INTECC) was antegradely introduced to pass as far as the dorsal artery. The occluded ATA was inflated using a SABER balloon catheter (Cordis; Fig 3, *A*), and the retrograde guide sheath was removed while being compressed with a Coyote balloon catheter (Boston; Fig 3, *B*). Next, IN.PACT Admiral drug-coated balloon catheters (Medtronic) were inflated in the proximal superficial femoral artery (Fig 3, *C*) and POPA (Fig 3, *D*). Good vascular patency and blood flow were achieved (Fig 3, *E* and *F*). The procedure time was 202 minutes, and the absorbed radiation dose and effective radiation dose were 0.2065 Gy and 29 μSv, respectively.

Dual antiplatelet therapy with 100 mg of aspirin and 75 mg of clopidogrel was conducted for 3 months, following clopidogrel monotherapy thereafter. Although the condition of the lower limb remained relatively stable following the EVT, the patient developed sudden acute limb ischemia on the same side 4 months later. While emergency endovascular intervention succeeded in establishing a one straight line of flow via posterior tibial artery, the lower limb gangrene worsened, and the patient finally died 5 months after that event.

## Discussion

An antegrade approach to the origin of the ATA, which branches sharply from a relatively large POPA with a preserved lumen, can sometimes be difficult, making a retrograde approach essential. Here, we report a case in which the procedure was limited to a retrograde approach, and a successful outcome was achieved using a series of strategies, including pedal angioplasty preceding distal puncture, sheath introduction, and second guide wire manipulation under IVUS marking.

First, a previous report demonstrated the successful IVUS-guided antegrade puncture of a stumpless posterior tibial occlusion.^3^ IVUS is usually useful for identifying the angiographically ambiguous entry of the branching vessel. However, despite our efforts, we were unable to detect the origin of the ATA using IVUS. Moreover, multiple anatomical and diagnostic imaging studies demonstrate that preprocedural assessment of the branching patterns of the POPA using CT angiography is crucial for planning EVT procedure.^4^^,^^5^ However, we had no preprocedural CT information in this case. Therefore, we promptly proceeded to the retrograde approach.

Several options are available for retrograde access, such as transcollateral, transpedal, or distal puncturing. As our case initially showed neither promising collateral arteries nor distal puncture sites, we had no choice but to select the transpedal approach. Although recent articles have demonstrated a transpedal retrograde wire just marker technique for below-the-knee occlusions with ambiguous proximal caps,^6^^,^^7^ we hesitated to use this technique because of the final requirement for antegrade preparation, even with this technique. To enhance retrograde guidewire maneuverability, retrograde access from the dorsal artery was established by puncturing after pedal artery angioplasty.^6^^,^^8^

Recently, multiple distal puncture sites have been shown to be safe and effective in EVT for lower-extremity arterial disease.^9^^,^^10^ However, the retrograde EVT system based on this type of distal puncture takes advantage of only microcatheter support, which limits the variation in retrograde EVT strategies. In contrast, there are several reports on tibiopedal arterial minimally invasive retrograde revascularization (TAMI),^11^ in which a 4F regular sheath is used as the vascular access route. Moreover, unlike the TAMI technique, the concept of transankle intervention (TAI) has emerged owing to the evolution of slender guide sheath devices.^12^ TAI can be used with various EVT devices including stents, whereas, only balloon angioplasty is available in TAMI, suggesting that EVT procedures can be completed using only a retrograde approach.

We retrogradely introduced a 3F guide sheath mainly for IVUS marking at the retrograde guidewire that deviated from the intraplaque to the subintimal space and successfully navigated the guidewire to the ostial ATA. Finally, we passed the ATA occlusion through the retrograde guidewire-crossing procedure, and the angiographic results were satisfactory after balloon angioplasty. However, clinical concerns regarding the long-term patency of the ATA ostium remain, owing to the possibility of passage through the subintimal space at the crossing point. Moreover, hemostatic management is crucial for both TAMI and TAI techniques, because these maneuvers pose a risk of occlusion of the punctured artery due to excessive compression. Therefore, we should avoid applying this maneuver to the only remaining infra-POPA. In our case, as the dorsal artery was occluded from the beginning, we considered guide sheath insertion to be sufficiently safe, even if arterial occlusion occurred after the procedure.

## Conclusions

Herein, we present a case of chronic limb-threatening ischemia with ambiguous occlusion of the ATA entry, in which stepwise modification of the EVT strategy was a critical determinant of successful revascularization. We must continue to improve the success rate of EVT for infrapopliteal lesions by developing treatment strategies while carefully examining the advantages and disadvantages of various retrograde approaches.

## Funding

None.

## Disclosures

None.

## References

[1] Schmidt A., Bakker O.J., Bausback Y., Scheinert D. (2017). The tibiopedal retrograde vascular access for challenging popliteal and below-the-knee chronic total occlusions: literature review and description of the technique. J Cardiovasc Surg (Torino).

[2] Conte M.S., Bradbury A.W., Kolh P. (2019). Global vascular guidelines on the management of chronic limb-threatening ischemia. Eur J Vasc Endovasc Surg.

[3] Halabi S., Al Hennawi H., Secemsky E.A., Palena M., Manzi M. (2025). Intravascular ultrasound guided puncture of stumpless tibial occlusions. Am J Cardiol.

[4] Oner S., Oner Z. (2020). Popliteal artery branching variations: a study on multidetector CT angiography. Sci Rep.

[5] Demirtaş H., Değirmenci B., Çelik A.O. (2016). Anatomic variations of popliteal artery: evaluation with 128-section CT-angiography in 1261 lower limbs. Diagn Interv Imaging.

[6] Buturak A., Tatli E., Erkin A. (2025). A novel troubleshooting technique for below-the-knee artery occlusions with proximal cap ambiguity: Transpedal Retrograde Wire Just Marker Technique. Vascular.

[7] Varim P., Buturak A., Cakmak A.C., Tatli E. (2025). Impact of transpedal retrograde wire just marker technique on revascularization of below the knee artery occlusions with ambiguous proximal caps in patients with Buerger's disease. Vascular.

[8] Sakaue Y., Nomura T., Ono K., Kikai M., Keira N., Tatsumi T. (2020). Pedal artery angioplasty for dorsal pedis artery puncture preceding endovascular treatment for occluded anterior tibial artery. Cardiovasc Interv Ther.

[9] Schmidt A., Bausback Y., Piorkowski M. (2019). Retrograde tibioperoneal access for complex infrainguinal occlusions: short- and long-term outcomes of 554 endovascular interventions. JACC Cardiovasc Interv.

[10] Ota I., Nomura T., Ono K., Sakaue Y., Shoji K., Wada N. (2023). Inframalleolar thrice distal puncture in a single endovascular treatment session for successful revascularization. CVIR Endovasc.

[11] Mustapha J.A., Saab F., McGoff T.N. (2020). Tibiopedal arterial minimally invasive retrograde revascularization (TAMI) in patients with peripheral arterial disease and critical limb ischemia. On behalf of the Peripheral Registry of Endovascular Clinical Outcomes (PRIME). Catheter Cardiovasc Interv.

[12] Nomura T., Wada N., Ono K., Shoji K. (2024). Bilateral transankle intervention as a critical determinant of successful revascularisation in complex peripheral arterial disease. EJVES Vasc Forum.

